# Targeted α-therapy using astatine (^211^At)-labeled PSMA1, 5, and 6: a preclinical evaluation as a novel compound

**DOI:** 10.1007/s00259-022-06016-z

**Published:** 2022-11-08

**Authors:** Tadashi Watabe, Kazuko Kaneda-Nakashima, Yoshifumi Shirakami, Yuichiro Kadonaga, Kazuhiro Ooe, Yang Wang, Hiromitsu Haba, Atsushi Toyoshima, Jens Cardinale, Frederik L. Giesel, Noriyuki Tomiyama, Koichi Fukase

**Affiliations:** 1 Department of Nuclear Medicine and Tracer Kinetics, Graduate School of Medicine, Osaka University, 2-2 Yamadaoka, Suita, Osaka, 565-0871 Japan; 2 Institute for Radiation Sciences, Osaka University, Osaka, Japan; 3 Core for Medicine and Science Collaborative Research and Education, Project Research Center for Fundamental Sciences, Graduate School of Science, Osaka University, Osaka, Japan; 4 Nishina Center for Accelerator-Based Science, RIKEN, Tokyo, Japan; 5 Department of Nuclear Medicine, Dusseldorf University, Düsseldorf, Germany; 6 Department of Radiology, Graduate School of Medicine, Osaka University, Osaka, Japan; 7 Department of Chemistry, Graduate School of Science, Osaka University, Osaka, Japan

**Keywords:** Theranostics, PSMA, Prostate cancer, Targeted α-therapy, Astatine

## Abstract

**Purpose:**

Targeted α-therapy (TAT) for prostate-specific membrane antigen (PSMA) is a promising treatment for metastatic castration-resistant prostate cancer (CRPC). Astatine is an α-emitter (half-life=7.2 h) that can be produced by a 30-MeV cyclotron. This study evaluated the treatment effect of ^211^At-labeled PSMA compounds in mouse xenograft models.

**Methods:**

Tumor xenograft models were established by subcutaneous transplantation of human prostate cancer cells (LNCaP) in NOD/SCID mouse. [^211^At]PSMA1, [^211^At]PSMA5, or [^211^At]PSMA6 was administered to LNCaP xenograft mice to evaluate biodistribution at 3 and 24 h. The treatment effect was evaluated by administering [^211^At]PSMA1 (0.40 ± 0.07 MBq), [^211^At]PSMA5 (0.39 ± 0.03 MBq), or saline. Histopathological evaluation was performed for the at-risk organs at 3 and 6 weeks after administration.

**Results:**

[^211^At]PSMA5 resulted in higher tumor retention compared to [^211^At]PSMA1 and [^211^At]PSMA6 (30.6 ± 17.8, 12.4 ± 4.8, and 19.1 ± 4.5 %ID/g at 3 h versus 40.7 ± 2.6, 8.7 ± 3.5, and 18.1 ± 2.2%ID/g at 24 h, respectively), whereas kidney excretion was superior in [^211^At]PSMA1 compared to [^211^At]PSMA5 and [^211^At]PSMA6. An excellent treatment effect on tumor growth was observed after [^211^At]PSMA5 administration. [^211^At]PSMA1 also showed a substantial treatment effect; however, the tumor size was relatively larger compared to that with [^211^At]PSMA5. In the histopathological evaluation, regenerated tubules were detected in the kidneys at 3 and 6 weeks after the administration of [^211^At]PSMA5.

**Conclusion:**

TAT using [^211^At]PSMA5 resulted in excellent tumor growth suppression with minimal side effects in the normal organs. [^211^At]PSMA5 should be considered a new possible TAT for metastatic CRPC, and translational prospective trials are warranted.

**Supplementary Information:**

The online version contains supplementary material available at 10.1007/s00259-022-06016-z.

## Introduction

Prostate cancer is one of the most common cancers worldwide. Approximately 1.41 million new cases of prostate cancer are diagnosed worldwide according to the Global Cancer Statistics 2020 [1]. Initial treatments for prostate cancer include surgery, radiation, and hormonal therapies, whereas active surveillance can be an option for low-grade malignancies without specific treatment [2]. Hormonal therapy is performed to treat recurrence after definitive therapy. However, prostate cancer finally becomes resistant as hormone-resistant cells can survive and have been selected during treatment, which is called castration-resistant prostate cancer (CRPC) [3]. The prognosis of metastatic CRPC is poor, and the median survival period is 9–13 months [4]. Although new androgen receptor inhibitors or chemotherapy using docetaxel or cabazitaxel can be provided to patients with non-metastatic or metastatic CRPC, some of them are progressive with a short doubling time of serum markers of prostate-specific antigen [5, 6].

The prostate-specific membrane antigen (PSMA) is an excellent target for theranostics. PSMA-positron emission tomography (PET) is useful for the detection of recurrent lesions, especially in biochemical recurrence after surgery or radiation therapy [7, 8]. PSMA uptake in recurrent lesions is usually remarkably high, and small metastases can be detected, which are difficult to detect using conventional computed tomography and bone scintigraphy [9]. For therapeutic applications, [^177^Lu]PSMA therapy has been recently approved by the US Food and Drug Administration in 2022 [10]. It significantly prolongs the overall survival of patients with metastatic CRPC compared with standard treatment alone [11]. Targeted α-therapy for PSMA is a promising therapy for metastatic CRPC [12]. [^225^Ac]PSMA is significantly effective even in refractory cases of [^177^Lu]PSMA therapy, despite the need to balance its dose due to its adverse effect of xerostomia [13].

[^225^Ac] has attracted attention for its labeling utility as a theranostic companion with [^68^Ga] and [^177^Lu]. However, its supply remains limited worldwide because its production requires nuclear fuel materials ([^229^Th] or [^232^Th]) or rare radioisotopes ([^226^Ra]) [14]. Astatine is an α-emitter (half-life = 7.2 h) that can be produced by a 30 MeV cyclotron with a reasonable cost and labeled to small molecules and peptides [15]. Sodium astatine ([^211^At]NaAt) and labeled amino acid analogs ([^211^At]PA and [^211^At]AAMT) are useful for the treatment of thyroid cancer, malignant glioma, pancreatic cancer, and malignant melanoma [16–18]. An investigator-initiated clinical trial using [^211^At]NaAt in patients with refractory thyroid cancer (ClinicalTrials.gov Identifier: NCT05275946) is in progress [19]. We also developed a novel labeling method using the substitution reaction of ^211^At with dihydroxyboryl groups [20]. Moreover, we developed a newly designed precursor based on the structure of [^18^F]PSMA-1007, which we believe is suitable for ^211^At-labeling [21]. In this study, we evaluated the characteristics of a novel ^211^At-labeled PSMA compound ([^211^At]PSMA5) and its therapeutic effect in a mouse xenograft model of prostate cancer and compared it with two closely related new derivatives, namely [^211^At]PSMA1 and [^211^At]PSMA6.

## Materials and methods

### Synthesis of [^211^At]PSMA1, [^211^At]PSMA5, and [^211^At]PSMA6

Precursor molecules of PSMA1, PSMA5, and PSMA6 were synthesized based on solid-phase peptide synthesis by Peptide Institute, Inc. (Osaka, Japan). ^211^At was produced by a nuclear reaction of ^209^Bi(α, 2n)^211^At using a cyclotron and purified by a dry distillation method, providing the aqueous solution of ^211^At (0.1–1 MBq/μL) [20].

**Fig. 1:**
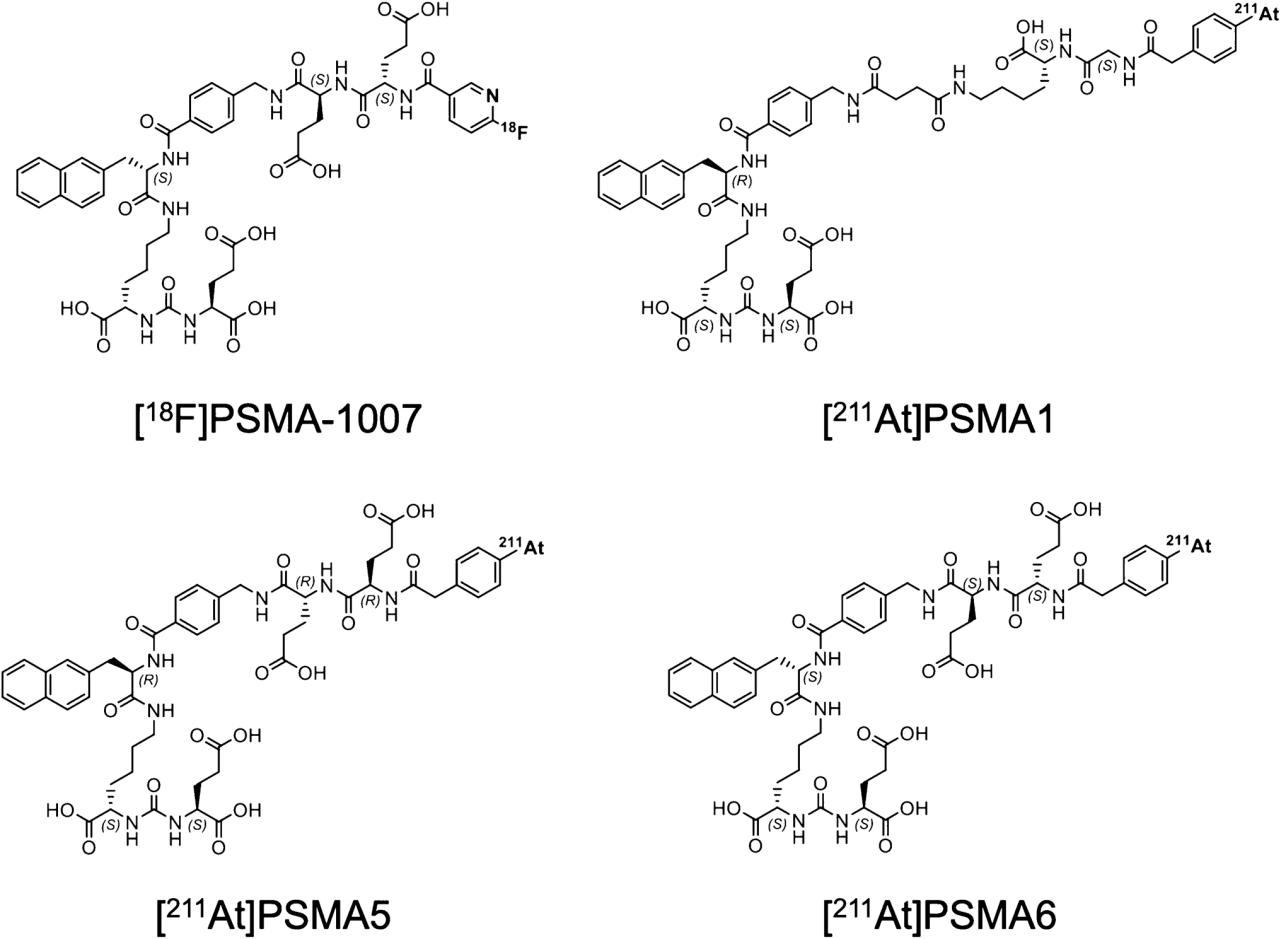
Molecular structures of [^18^F]PSMA-1007, [^211^At]PSMA1, [^211^At]PSMA5, and [^211^At]PSMA6

^211^At-labeled PSMA1, PSMA5, and PSMA6 were synthesized by the substitution reaction of ^211^At with the dihydroxyboryl groups introduced to the corresponding precursor molecules, as described in a previous paper [20]. Twenty microliters of 0.1 mg/mL PSMA1, PSMA5, or PSMA6 solution (containing 7% (w/v) sodium hydrogen carbonate was mixed with 1–40 MBq (1–100 μL) of aqueous solution of ^211^At. Subsequently, 20–40 μL of 0.1 mol/L KI solution was added to the mixture, which was allowed to react for 45 min at 80 °C. The molecular structures of [^211^At]PSMA1, [^211^At]PSMA5, and [^211^At]PSMA6 are shown in Fig. 1.

The crude reaction mixture of [^211^At]PSMA1, [^211^At]PSMA5, or [^211^At]PSMA6 was purified by solid-phase extraction (SPE). The mixture was loaded onto an Oasis HLB cartridge (Waters, Milford, US), and the cartridge was rinsed with 1 mL of aqueous solution of sodium hydrogen carbonate (7% (w/v)). [^211^At]PSMA1, [^211^At]PSMA5, and [^211^At]PSMA6 trapped in the cartridge were eluted with 1 mL of 20% (v/v) ethanol. The radiochemical yields of [^211^At]PSMA1, [^211^At]PSMA5, and [^211^At]PSMA6 were not less than 60% (radioactivity decay corrected), and the radiochemical purity of the products was greater than 96% after the SPE purification. Molar activities of the radioligands were 175–190 MBq/nmol. In the treatment experiments, the mass was approximately 0.002 μg per animal for 0.40 MBq of [^211^At]PSMA1 or [^211^At]PSMA5 solution. In a biodistribution study, the mass was approximately 0.0005–0.0006 μg when [^211^At]PSMA1, 5 or 6 was administered at 0.10–0.12 MBq/mouse.

### In vitro cellular uptake analysis

Human prostate cancer cell lines, prostatic carcinoma-3 (PC-3) (low expression of PSMA), and lymph node carcinoma of the prostate (LNCaP) (high expression of PSMA) were obtained from the RIKEN Cell Bank (Tsukuba, Japan). Cells were maintained in a culture medium, Roswell Park Memorial Institute 1640 medium (FUJIFILM Wako Pure Chemical Corporation, Osaka, Japan), supplemented with 10% heat-inactivated fetal bovine serum (Gibco) and 1% penicillin-streptomycin (FUJIFILM Wako Pure Chemical). The medium for LNCaP was supplemented with 1% sodium pyruvate (FUJIFILM Wako Pure Chemical) in a culture medium. Cells were seeded in 24-well plates (5 × 10^4^/well) and cultured for 2 days. After washing twice with phosphate-buffered saline (PBS) (−), the culture medium was changed to Hanks’ balanced salt solution (+). After treatment with [^211^At]PSMA1 or [^211^At]PSMA5 (approximately 30–50 kBq/well), cells were washed twice with PBS (–). After washing, all cells were lysed with 0.1 N sodium hydroxide, and the radioactivity of the cells was calculated using a 2480 Wizard^2^ γ counter (Perkin Elmer, MA, USA). Protein levels were measured using a plate reader (MultiScan FC, Thermo Fisher) and the BCA Protein Assay Kit (FUJIFILM Wako Pure Chemical). Uptake (%uptake/mg protein) was compared between PC-3 and LNCaP cells at 30 min after incubation with [^211^At]PSMA1 or [^211^At]PSMA5.

### Preparation of xenograft models

Non-obese diabetic/severe combined immunodeficiency (NOD/SCID) mice (5 weeks old, male) were purchased from Charles River Japan, Inc. (Atsugi, Japan). LNCaP cells were suspended in a 1:1 mixture of medium and Matrigel (Corning, USA), subcutaneously implanted into the unilateral flank of the mice (approximately 6–10 × 10^6^ cells), and used approximately 5 weeks later (range, 4–8 weeks). Institute of Cancer Research (ICR) mice (6 weeks old, male) were purchased from Japan SLC, Inc. (Shizuoka, Japan) and used as a non-tumor-bearing cohort for the evaluation of biodistribution and histology.

Euthanasia was performed under deep anesthesia using isoflurane inhalation. The criteria for euthanasia were as follows: (1) animals showed signs of intolerable suffering, (2) a significant decrease in activity or a marked decrease in food and water intake was observed, (3) the tumor size reached 2 cm in diameter, and (4) the observation period ended.

### Biodistribution of [^211^At]PSMA1, [^211^At]PSMA5, and [^211^At]PSMA6

LNCaP xenograft mice (body weight = 18.8 ± 3.0 g, *n* = 25) and normal ICR mice (body weight = 33.0 ± 1.3 g, *n* = 12) were used to evaluate biodistribution after the administration of [^211^At]PSMA solutions ([^211^At]PSMA1, 0.12 ± 0.10 MBq; [^211^At]PSMA5, 0.11 ± 0.04 MBq; and [^211^At]PSMA6, 0.11 ± 0.02 MBq, 0.0005–0.0006 μg). High PSMA expression was already confirmed in LNCaP xenografts in our previous study [22]. The brain, thyroid, salivary gland, lung, heart, liver, spleen, pancreas, stomach, small intestine, colon, kidney, bone, testis, blood, urine, feces, and tumor were excised and weighed to evaluate biodistribution after euthanasia at 3 and 24 h after administration. Urine excretion was determined from absorption to filter paper or by urine collection in the cage, and feces were collected from the cage. Radioactivity was measured using a 2480 Wizard^2^ γ counter. The detection efficiency for ^211^At with the γ counter was calibrated by measurement of the ^211^At source whose radioactivity was determined with a Ge semiconductor detector (BE2020, Mirion Technologies (Canberra), Connecticut, USA). Uptake was calculated as the percentage of injected dose (%ID).

Planar imaging was performed using a γ camera system (E-cam, Siemens) at 3 and 24 h after administration, targeting the X-rays emitted from the daughter nuclide ^211^Po (energy window: 79 keV ± 20%) [16]. Image analysis was performed by setting the regions of interest in the tumor and kidneys using AMIDE software (version 1.0.4).

### Evaluation of treatment effect of [^211^At]PSMA5 and [^211^At]PSMA1

LNCaP xenograft mice (body weight = 21.8 ± 5.98 g) were administered [^211^At]PSMA5 (0.39 ± 0.03 MBq, 0.002 μg, *n* = 12), [^211^At]PSMA1 (0.40 ± 0.07 MBq, 0.002 μg, *n* = 5), or saline (*n* = 10). The treatment dose was based on that used in our previous study, in which 0.4MBq of [^211^At]NaAt showed sufficient therapeutic effect without significant toxicity [16, 23]. Non-radiolabeled PSMA5 (high mass, 0.02 μg; low mass, 0.002 μg) and PSMA1 (high mass, 0.02 μg; low mass, 0.002 μg) were also administered to LNCaP xenograft mice (each *n* = 3) and compared with saline-injected mice (*n* = 3) to assess the absence of antitumor effect by the PSMA compound itself. Tumor sizes (mm^3^) were measured using a caliper, calculated using the following elliptical sphere model equation, and compared between injected mice and controls. The body weight (g) was also monitored.

### Evaluation of side effects

After euthanasia, the thyroid, salivary gland, stomach, small intestine, spleen, and kidney were excised from normal ICR mice (body weight = 32.0 ± 2.0 g, *n* = 10) 8 weeks after the administration of [^211^At]PSMA5 (0.33 ± 0.003 MBq) or [^211^At]PSMA6 (0.35 ± 0.023 MBq). The thyroid, salivary gland, and kidney were excised from normal ICR mice (body weight = 33.5 ± 1.6 g, *n* = 4) 2 weeks after the administration of [^211^At]PSMA5 (high-dose: 1.06 ± 0.06 MBq). The salivary gland, stomach, and kidney were excised from LNCaP xenograft mice (*n* = 14, body weight = 21.8 ± 5.98 g) 3 and 6 weeks after the administration of [^211^At]PSMA5 (0.37 ± 0.02 MBq). The excised organs and tissues were fixed in a 10% neutral buffered formalin solution. After fixation, paraffin sections were prepared and stained with hematoxylin and eosin. Specimens were evaluated using an integrated microscope (BZ-X810; Keyence Corporation, Osaka, Japan). Histological evaluation was performed by a toxicopathology specialist with the support of KAC Co. Ltd. (Kyoto, Japan).

Plasma was obtained by centrifuging a portion of the blood sample collected at the time of euthanasia and was measured using a dry clinical chemistry analyzer (SPOTCHEM D-00 QR D-02; ARKRAY, Inc., Kyoto, Japan). Blood urea nitrogen (BUN) and creatinine (Cre) levels were also measured. Cre values less than 0.2 were considered 0.2 in the statistical analysis. Urine analysis was also performed using urinalysis test strips (Multistix Ames 2820, Siemens Healthcare, Tokyo, Japan) during the observation period in normal ICR mice after the administration of [^211^At]PSMA5.

### Statistical analyses

Comparisons between two groups were performed using the unpaired *t*-test in SPSS (version 25.0, IBM Corp., Armonk, NY, USA). For multiple comparisons among the three groups, Bonferroni correction was performed. Differences were considered statistically significant at *P* < 0.05.

## Results

**Fig. 2:**
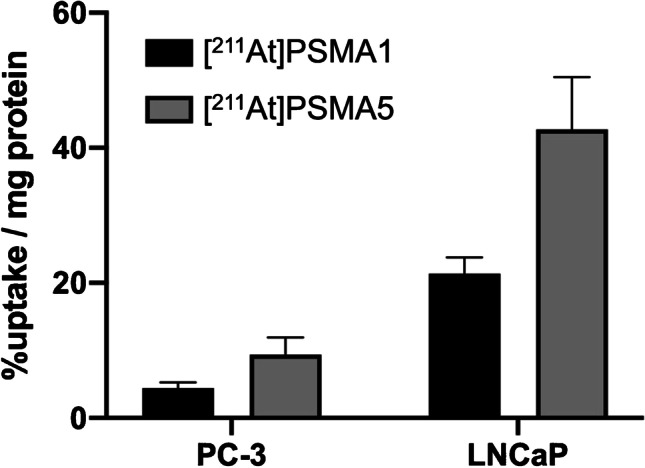
Cellular uptakes of [^211^At]PSMA1 and [^211^At]PSMA5 in PC-3 cells (low expression of PSMA) and LNCaP cells (high expression of PSMA). Data are expressed as mean ± standard deviation

In the cellular uptake analysis, [^211^At]PSMA1 and [^211^At]PSMA5 were more highly incorporated into LNCaP cells with high PSMA expression than in PC-3 cells with low PSMA expression (Fig. 2), suggesting PSMA-mediated uptake of both compounds. Moreover, [^211^At]PSMA5 showed higher uptake than [^211^At]PSMA1.

**Fig. 3:**
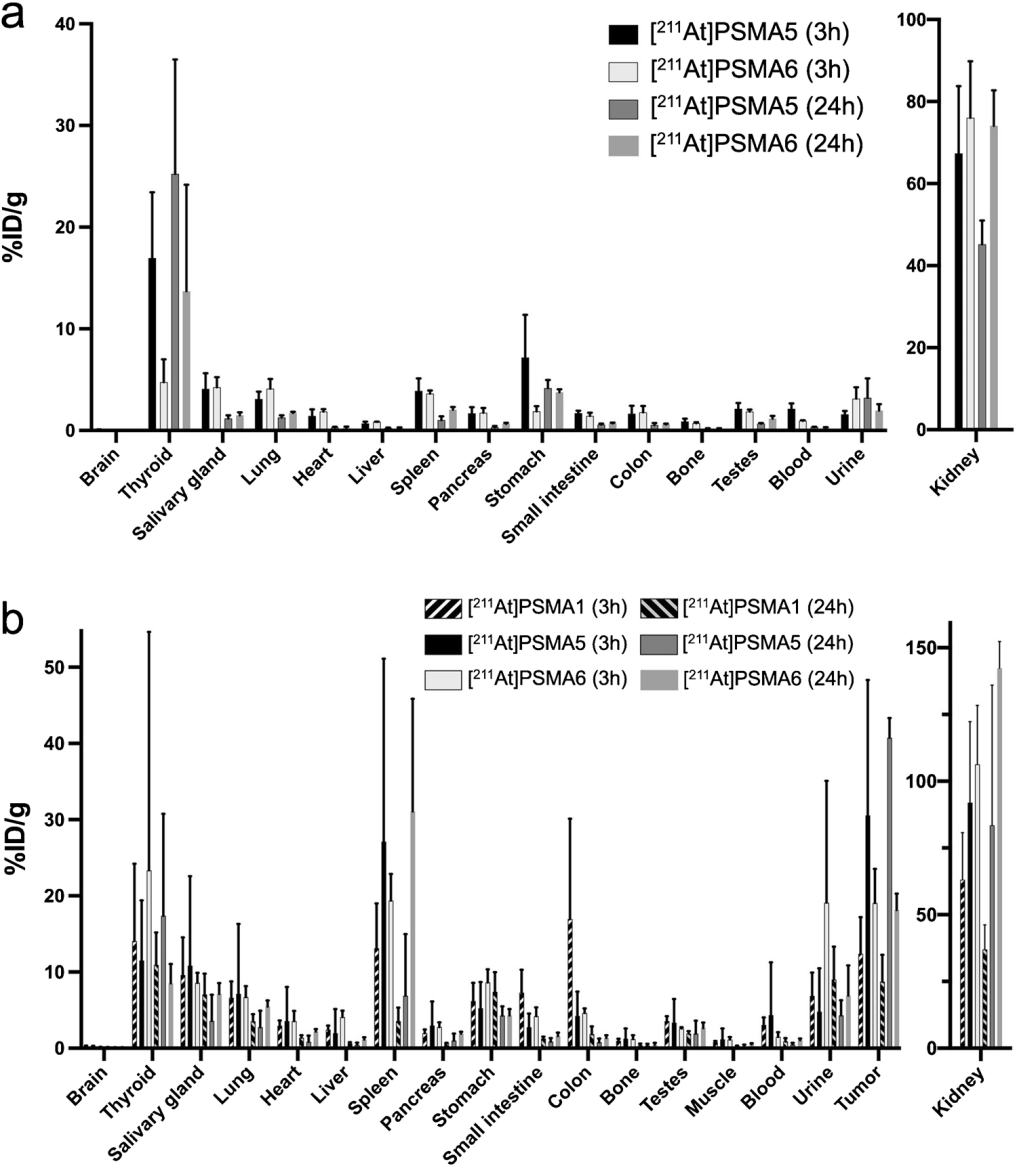
**a** Biodistribution of [^211^At]PSMA5 and [^211^At]PSMA6 in normal ICR mice. **b** Biodistribution of [^211^At]PSMA1, [^211^At]PSMA5, and [^211^At]PSMA6 in LNCaP xenograft model (NOD/SCID mice). Data are expressed as mean ± standard deviation. Total excretion (%ID) of [^211^At]PSMA1 and [^211^At]PSMA5 were 0.49 ± 0.37% and 8.26 ± 5.00 % at 3h and 14.7 ± 10.0% and 15.33 ± 6.3% at 24h in the urine, and 14.0 ± 4.8% and 35.2 ± 9.2% at 24h in the feces, respectively. Total excretion (%ID) of [^211^At]PSMA6 at 24h were 6.20 ± 1.51% in the urine, and 9.78 ± 0.79% in the feces, respectively

**Fig. 4:**
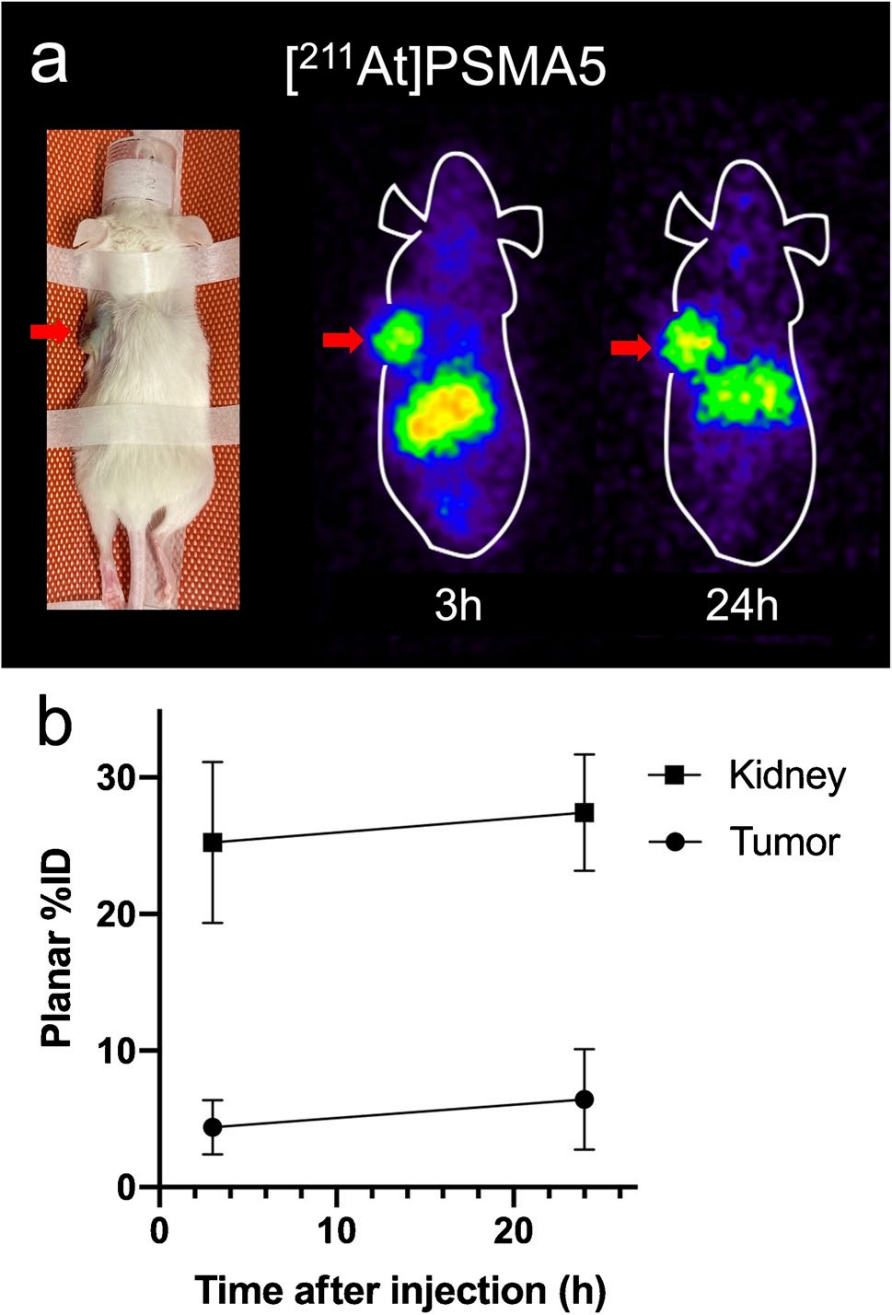
**a** Planar images of [^211^At]PSMA5 in LNCaP xenograft mice. **b** Region-of-interest analysis of [^211^At]PSMA5 in kidney and tumor

As shown in Fig. 3, kidney uptake was similar at 3 h post-injection in both NOD/SCID and ICR mice between [^211^At]PSMA5 and [^211^At]PSMA6, but [^211^At]PSMA6 showed higher retention in the kidney 24 h post-injection than [^211^At]PSMA5. [^211^At]PSMA5 showed higher tumor uptake than [^211^At]PSMA6. Therefore, [^211^At]PSMA5 showed better tumor-to-kidney uptake than [^211^At]PSMA6 did. [^211^At]PSMA5 showed better tumor retention compared to [^211^At]PSMA1 and [^211^At]PSMA6 (30.6 ± 17.8, 12.4 ± 4.8 and 19.1 ± 4.5%ID/g at 3 h versus 40.7 ± 2.6, 8.7 ± 3.5 and 18.1 ± 2.2%ID/g at 24 h, respectively), whereas kidney excretion was superior in [^211^At]PSMA1 compared to [^211^At]PSMA5 and [^211^At]PSMA6. The planar images of [^211^At]PSMA5 are shown in Fig. 4. High uptake was observed in the tumor xenografts and kidneys at 3 and 24 h post-injection.

**Fig. 5:**
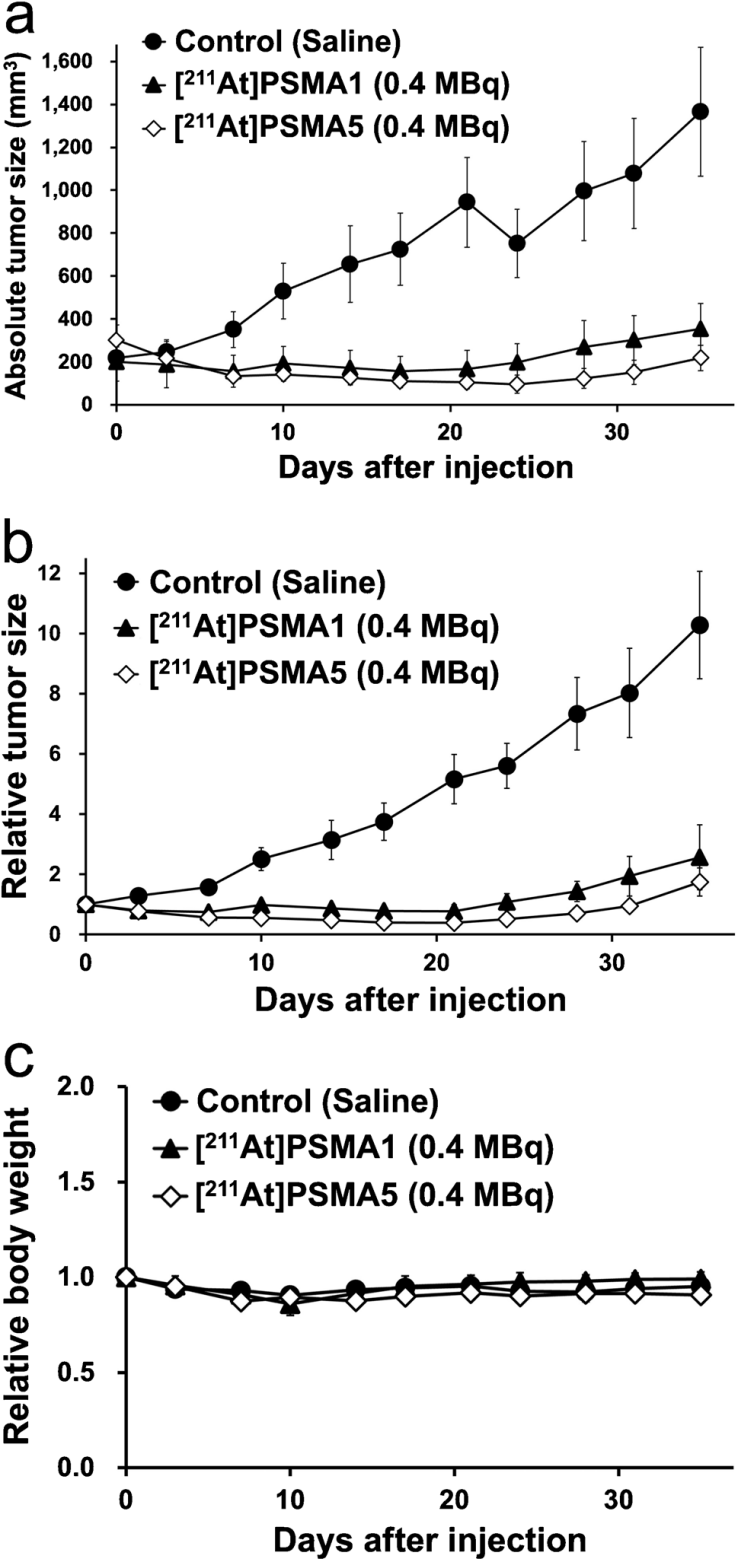
**a**, **b** Tumor size and **c** changes in body weight in LNCaP xenograft mice after the single administration of [^211^At]PSMA1 (0.4 MBq, *n* = 5), [^211^At]PSMA5 (0.4 MBq, *n* = 12), or control (saline, *n* = 10)

Regarding the treatment effect, excellent tumor growth suppression was observed in LNCaP xenograft after the administration of [^211^At]PSMA5 (Fig. 5a, b). [^211^At]PSMA1 also showed a good treatment effect, but it showed relatively larger tumor size than [^211^At]PSMA5 did. No significant changes in body weight were observed among the three groups (Fig. 5c).

**Fig. 6:**
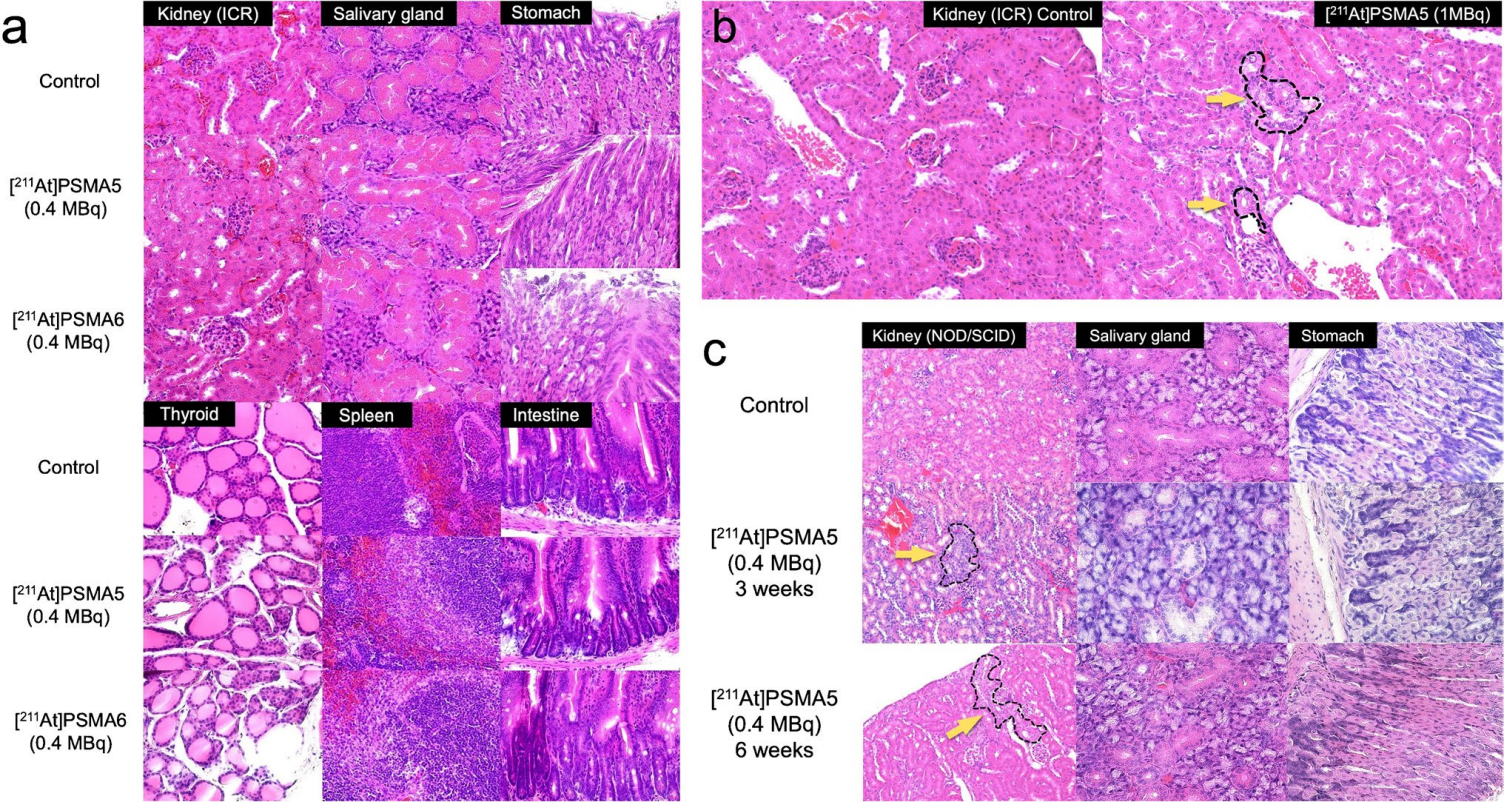
**a** H&E staining of the kidney, salivary gland, stomach, thyroid, spleen, and small intestine 8 weeks after the administration of [^211^At]PSMA5 (0.4 MBq) or [^211^At]PSMA6 (0.4 MBq) in normal ICR mice. **b** H&E staining of the kidney 2 weeks after the administration of [^211^At]PSMA5 (high-dose: 1 MBq) in normal ICR mice. **c** H&E staining of the kidney, salivary gland, and stomach 3 and 6 weeks after the administration of [^211^At]PSMA5 (0.4 MBq) in LNCaP xenograft mice. Dotted area with yellow arrows indicated basophilic tubules in the cortical area, suggesting regenerated tubules

In the histopathological evaluation, no significant changes were observed in the kidney parenchyma, salivary gland, stomach, thyroid, spleen, and small intestine of normal ICR mice 8 weeks after the administration of [^211^At]PSMA5 or [^211^At]PSMA6 (0.4 MBq) (Fig. 6a). In one out of four ICR mice administered [^211^At]PSMA5 (1 MBq), regenerated tubules were observed in the cortical area (Fig. 6b). In NOD/SCID mice, regenerated tubules were observed in the kidneys 3 and 6 weeks after administration in LNCaP xenograft mice (Fig. 6c). No significant changes were observed in the salivary glands or stomach.

## Discussion

In this study, we evaluated the novel ^211^At-labeled PSMA compounds, [^211^At]PSMA1, [^211^At]PSMA5, and [^211^At]PSMA6. These three PSMA analogs were designed and synthesized based on the structure of [^18^F]PSMA-1007 as a scaffold [7–9]. We introduced an aryl boronic acid for the ^211^At labeling of our PSMA precursors instead of *N*,*N*,*N*-trimethyl-2-pyridinaminium moiety for ^18^F labeling. The three PSMA analogs have different amino acid residues in their side chains, Gly-Lys, ©-G©(R)-Glu, and (S)-Glu-(S)-Glu in PSMA1, PSMA5, and PSMA6, respectively. We evaluated the effects of the differences in amino acid residues on the properties of tumor retention, biodistribution, and in vivo treatment effects.

At first, we compared [^211^At]PSMA5 and [^211^At]PSMA6 by performing a biodistribution study and histological evaluation of the major organs. The results showed that tumoral uptake at 3 h was higher in [^211^At]PSMA5 than in [^211^At]PSMA6, whereas kidney retention was higher in [^211^At]PSMA6 compared to [^211^At]PSMA5. Therefore, we selected [^211^At]PSMA5 as the main candidate compound. Next, we developed a new compound namely [^211^At]PSMA1 and compared its cellular uptake, biodistribution, and treatment effect in xenograft mice with [^211^At]PSMA5. The result showed that [^211^At]PSMA5 exhibited the best tumor retention and excellent tumor growth suppression in LNCaP xenograft models compared to [^211^At]PSMA1. As no significant change in tumor growth was observed in the non-radiolabeled PSMA compounds (Supplementary Fig. S1), the antitumor effect of [^211^At]PSMA5 was attributed to the α-particle emission from ^211^At.

In the cellular uptake analysis, [^211^At]PSMA5 showed higher uptake than [^211^At]PSMA1, corresponding to the in vivo uptake in tumor xenograft models. In addition, uptake was higher in LNCaP cells than in PC-3 cells, suggesting that PSMA mediates the uptake of [^211^At]PSMA1 and [^211^At]PSMA5. In the whole-body biodistribution of [^211^At]PSMA compounds, the kidneys showed remarkably high uptake, similar to the other PSMA compounds, reflecting PSMA expression in the proximal tubule and urine excretion [24]. Mild uptake was observed in the thyroid, spleen, and stomach. These uptakes were the physiological uptake of sodium astatide (NaAt), suggesting dehalogenation of [^211^At] from [^211^At]PSMA5/6 [16]. [^211^At]PSMA5 was subjected to slow deastatination in mice, resulting in not more than 1.0% of the injected doses of the metabolites, including astatide ions, to be present in urine at 3 h after injections of the agents. In the thyroid, variable uptake was observed, possibly due to the excision of the surrounding tissues, including the trachea, which influenced the variability in organ weight. Although we did not observe histological changes in the thyroid, it can be a risk organ for radioligand therapy using [^211^At]-labeled compounds. We have an option to use iodine blocking in clinical applications to protect the thyroid by inhibiting its uptake [17, 25]. Furthermore, if we increase the injected dose, the non-radiolabeled mass in the solution also increases proportionately to the radioactivity. This may affect the biodistribution of the [^211^At]-labeled compound due to competitive binding.

In the histopathological evaluation of the kidneys after administration of [^211^At]PSMA5, regenerated tubules were observed in the cortical area in all NOD/SCID mice, although most of them showed mild changes. These changes were not observed in the same dose group (0.4 MBq) of ICR mice, and only one out of four mice in the high-dose group (1 MBq) experienced these changes. Regenerated tubules are characterized by tubule basophilia, nuclear crowding, and increased mitoses. They were reported to occur as a reparative response to previous degeneration and/or necrosis of renal tubular epithelium [26]. It was also observed in the chronic phase after internal irradiation with α-emitting daughter nuclides of ^225^Ac [27]. This may be due to the radiation-induced toxicity of [^211^At]PSMA5, as PSMA expression was observed in the proximal tubule of the kidney [24]. In a previous study by Pomper et al., late nephrotoxicity was reported in PSMA-targeted ^211^At-labeled α-particle radiotherapy [28]. They showed its uptake in the cortical area of the kidney by α-camera imaging, subcortical atrophy, and degenerative loss of proximal tubules after treatment with ^211^At-6 (1.5 MBq). They also reported that all animals treated with 1.5 MBq developed proteinuria 1–2 months after treatment, and animals treated with 37 kBq developed mild proteinuria that was later resolved. In our study, we did not observe proteinuria or increased BUN and Cre levels 8 weeks after the administration of [^211^At]PSMA5, although uptake in the kidneys was similar in ICR mice compared with the report (60–70%ID/g at 1–18 h after administration) (Supplementary Fig. S2). However, chronic long-term kidney toxicity requires further evaluation from the perspective of future translation [29]. We aim to perform an extended single-dose toxicity study with three doses, including the evaluation of hematological toxicity (acute and recovery phases) and long-term chronic kidney toxicity (additional group) based on our previous report [23].

Xerostomia is the most common side effect of clinical targeted α-therapy using [^225^Ac]PSMA-617, since PSMA expression was also observed in the salivary gland [12, 13]. However, we did not observe any histological abnormalities in salivary glands. There might be a species difference, since the uptake in the salivary glands was not significantly higher in mice than in humans. We need to carefully monitor the toxicity in salivary glands in future clinical applications.

In clinical translation, species differences are sometimes observed. In [^18^F]PSMA-1007 PET, high urine excretion was observed in mice, but its excretion was minimal in humans [21, 30]. Diagnostic PET for evaluating prostate cancer recurrence has better detectability without excretion in the urinary tract [9]. However, for therapeutic applications, rapid urine excretion is ideal for reducing the absorbed dose in the kidneys. Although a continuous high uptake of [^211^At]PSMA5 was observed in the kidneys, no serious toxicity was observed in this study. The radioactivity in the kidneys was presumably from the intact molecule since most of the radioactivity observed in the blood and urine was from intact molecules at 3 h post injection of the agent. Kidney retention may not be a significant problem for targeted α-therapy using ^211^At because of its short physical half-life (7.2 h). However, in humans, it has been hypothesized that renal function tends to decline due to past cancer treatments, and the initial clinical dose of [^211^At]PSMA5 should be determined carefully.

This study had some limitations. First, we evaluated the treatment effect in the LNCaP model using a single-dose administration. Repeated administration or dose escalation should be evaluated in future studies to mimic clinical situations and to define a minimum or maximum effective dose with a longer observation period. Second, we evaluated toxicity mainly using histological analysis. Hematological toxicity, including myelosuppression, should be evaluated in greater detail. Third, a detailed evaluation of whole-body distribution at multiple time points is essential for a precise dosimetric approach. Pharmacokinetic studies should be conducted in the future to perform precise estimation of absorbed doses and comparison with histological abnormalities.

## Conclusion

[^211^At]PSMA5 exhibited excellent tumor growth suppression in xenograft models of prostate cancer, with minimal side effects. [^211^At]PSMA5 could be a new possible targeted α-therapy for prostate cancer, specifically metastatic CRPC, and future translational prospective trials are warranted.

## Supplementary Information

Below is the link to the electronic supplementary material.

Supplementary file1 (DOCX 615 kb)

## Data Availability

Data available on request.
