# Supplementary material for: Targeted α-therapy using astatine (211At)-labeled PSMA1, 5, and 6: a preclinical evaluation as a novel compound
Source: Eur J Nucl Med Mol Imaging. 2022 Nov 8;50(3):849–58. doi: 10.1007/s00259-022-06016-z (PMC9852121; doi:10.1007/s00259-022-06016-z)
Supplement: Supplementary file 1 — Supplementary file1 (DOCX 615 kb) [file 259_2022_6016_MOESM1_ESM.docx]

**Supplementary information**

**Targeted alpha therapy using astatine (^211^At)-labeled PSMA1,5 and 6: a preclinical evaluation as a new novel compound**

Tadashi Watabe^1,2^, Kazuko Kaneda-Nakashima^2,3^, Yoshifumi Shirakami^2^, Yuichiro Kadonaga^2^, Kazuhiro Ooe^1,2^, Yang Wang^4^, Hiromitsu Haba^4^, Atsushi Toyoshima^2^, Jens Cardinale^5^, Frederik L. Giesel^5^, Noriyuki Tomiyama^2,6^, Koichi Fukase^7^

^1^Department of Nuclear Medicine and Tracer Kinetics, Graduate School of Medicine, Osaka University

^2^Institute for Radiation Sciences, Osaka University

^3^Core for Medicine and Science Collaborative Research and Education, Project Research Center for Fundamental Sciences, Graduate School of Science, Osaka University

^4^Nishina Center for Accelerator-Based Science, RIKEN

^5^Department of Nuclear Medicine, Dusseldorf University

^6^Department of Radiology, Graduate School of Medicine, Osaka University

^7^Department of Chemistry, Graduate School of Science, Osaka University

Corresponding and first author:

Tadashi Watabe (Assistant Professor)

2-2 Yamadaoka, Suita, Osaka 565-0871, Japan

Tel: +81-6-6879-3461

Fax: +81-6-6879-3469

E-mail: [watabe@tracer.med.osaka-u.ac.jp](mailto:watabe@tracer.med.osaka-u.ac.jp)

ORCID: 0000-0001-8658-2395


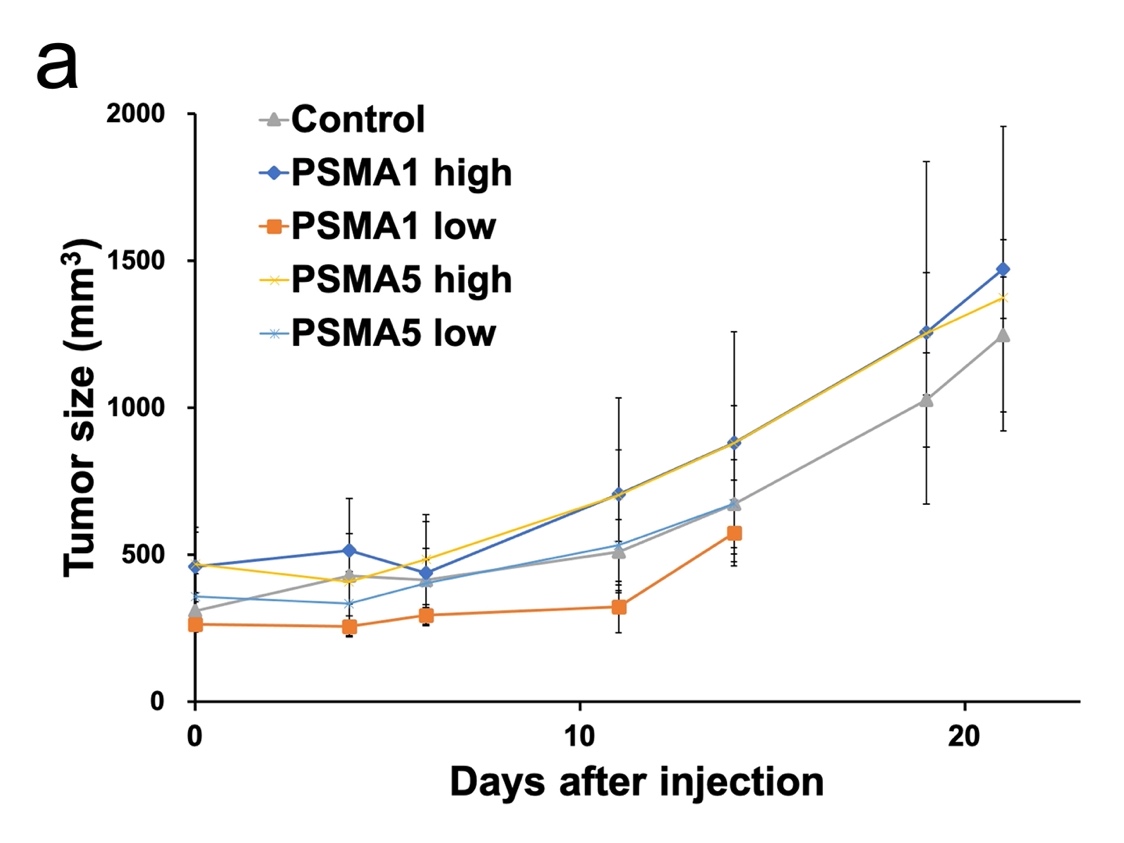


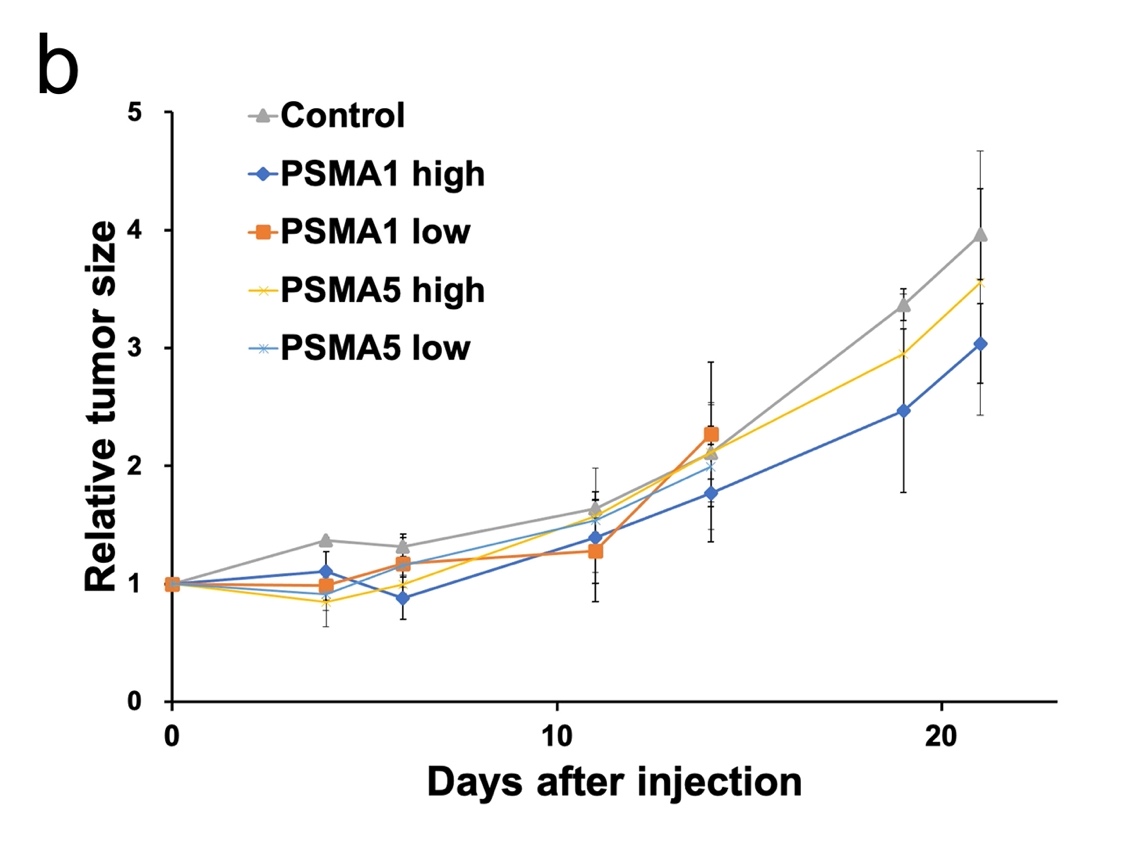


**Supplementary Fig. S1.** Tumor growth curve after the single administration of non-radiolabeled PSMA1, PSMA5, and control (saline): (a) actual tumor size and (b) relative tumor size. High and low correspond to the peptide dose of 10 MBq and 1 MBq of [^211^At]PSMA1 and [^211^At]PSMA5, respectively.


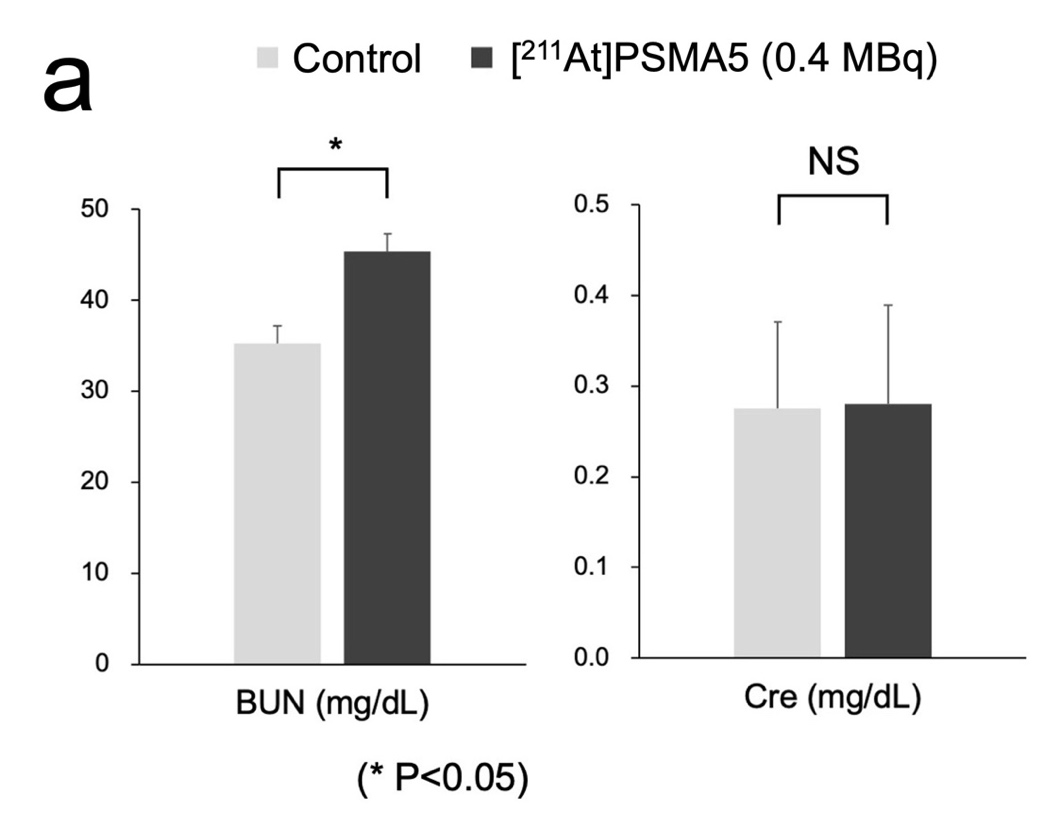


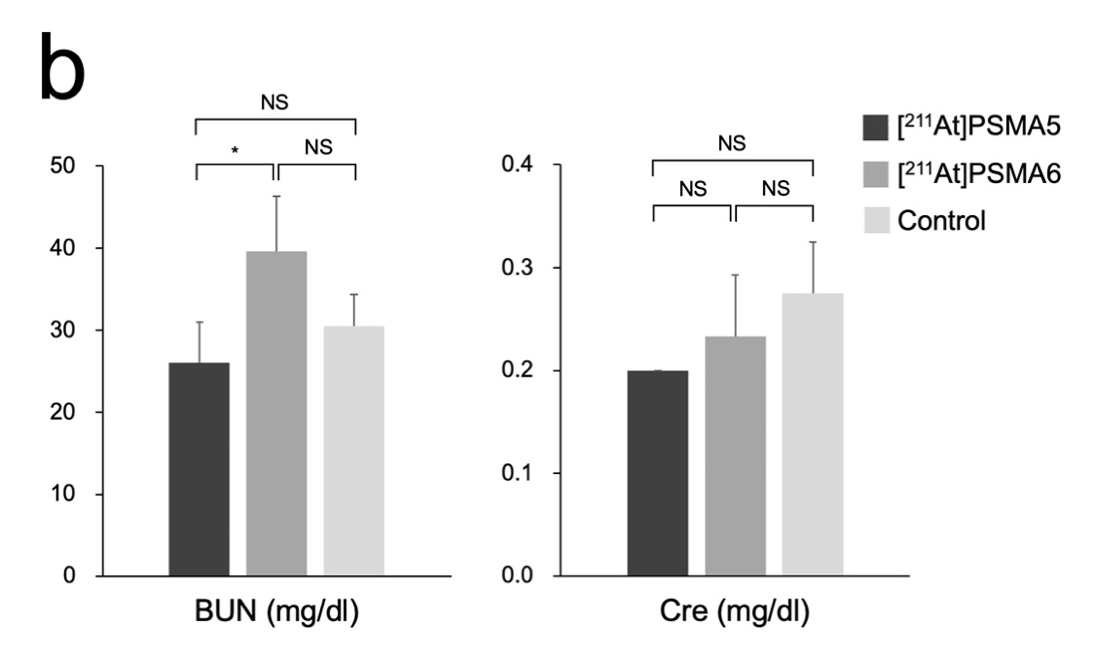


**Supplementary Fig. S2.** Evaluation of renal function using blood sample (a) 3 weeks after the administration of [^211^At]PSMA5 (0.4 MBq) in NOD/SCID mice and (b) 8 weeks after the administration of [^211^At]PSMA5 (0.4 MBq) and [^211^At]PSMA6 (0.4 MBq) in ICR mice (^*^p < 0.05; NS, not significant).

1. **Materials**

Structures of precursor molecules for ^211^At labeling, PSMA-1, PSMA-5 and PSMA-6, are shown in Supplemental Figure 1

PSMA-1

PSMA-5 　　　　　　　　　　　　　 PSMA-6

**Supplemental Fig. S3.** Chemical structures of precursor molecules

Chemical names of the precursors are as follows.

**PSMA-1:**

(((S)-5-((R)-2-(4-((4-(((R)-5-(2-(2-(4-boronophenyl)acetamido)acetamido)-5-carboxypentyl)amino)-4-oxobutanamido)methyl)benzamido)-3-(naphthalen-2-yl)propanamido)-1-carboxypentyl)carbamoyl)-L-glutamic acid

PSMA-2:(((S)-5-((R)-2-(4-(((R)-2-((R)-2-(4-(((R)-5-(2-(2-(4-boronophenyl)acetamido)acetamido)-5-carboxypentyl)amino)-4-oxobutanamido)-4-carboxybutanamido)-4-carboxybutanamido)methyl)benzamido)-3-(naphthalen-2-yl)propanamido)-1-carboxypentyl)carbamoyl)-L-glutamic acid

**PSMA-5:**

(((S)-5-((R)-2-(4-(((R)-2-((R)-2-(2-(4-boronophenyl)acetamido)-4-carboxybutanamido)-4-carboxybutanamido)methyl)benzamido)-3-(naphthalen-2-yl)propanamido)-1-carboxypentyl)carbamoyl)-L-glutamic acid

**PSMA-6:**

(((S)-5-((R)-2-(4-(((S)-2-((S)-2-(2-(4-boronophenyl)acetamido)-4-carboxybutanamido)-4-carboxybutanamido)methyl)benzamido)-3-(naphthalen-2-yl)propanamido)-1-carboxypentyl)carbamoyl)-L-glutamic acid

**HPLC profiles of the precursors**

Conditions:

HPLC system: Shimadzu LC-20

Column: C18 MSII, 150 x 4.6 mm

Temperature: 30°C

Detector: UV 226 nm

Injection volume: 50 µL

Sample concentration: 1 µg/mL

Solvent: A) 5mmol/L Tetrabuthylammonium phosphate, pH7.5

B) Acetonitrile

Time solvent A solvent B

Gradient: 0 - 5 min 70% 30%

5 - 30 min 70% to 20% 30% to 80%

Chromatograms:

**Supplemental Fig. S4.** HPLC analysis of precursors.

Radio-TLC:

**Crude products** **Purified products**

^211^At-PSMA-1 (RCY: 84.8%) ^211^At-PSMA-1 (RCY: 98.0%)


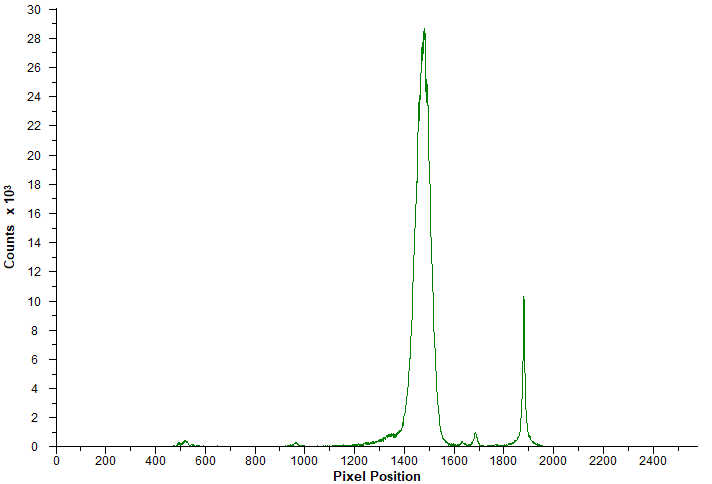

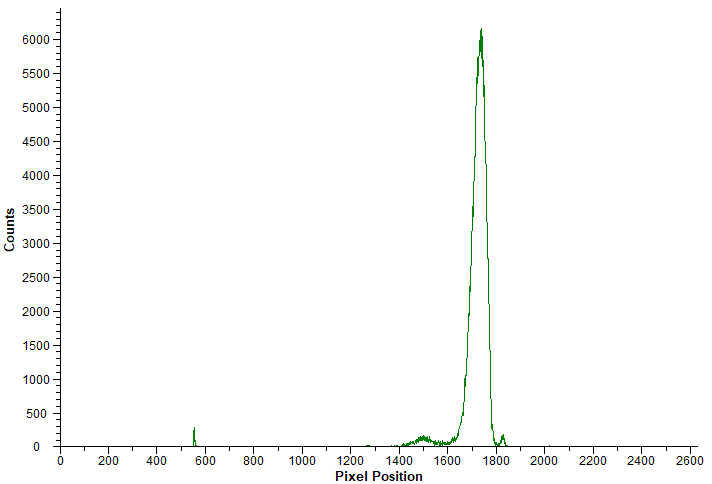


^211^At-PSMA-5 (RCY: 78.6%) ^211^At-PSMA-5 (RCY: 99.8%)


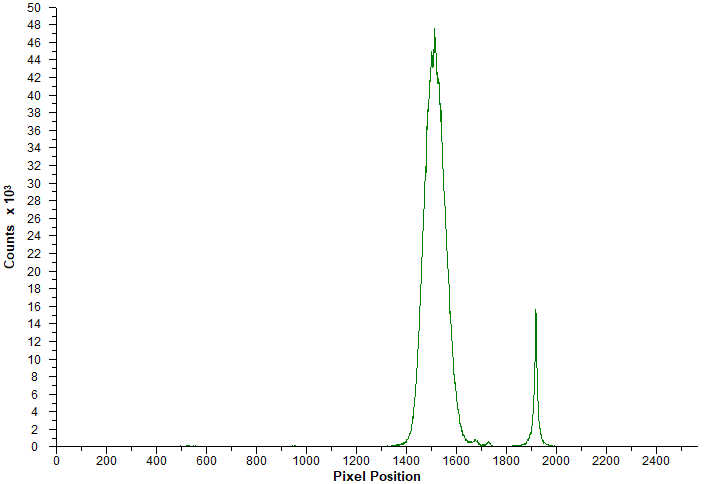

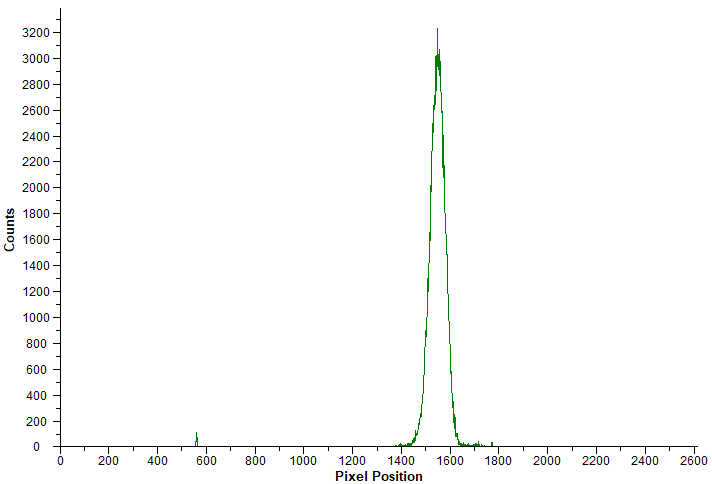


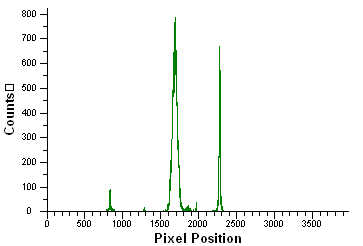
^211^At-PSMA-6 (RCY: 72.8%) ^211^At-PSMA-6 (RCY: 72.8%)


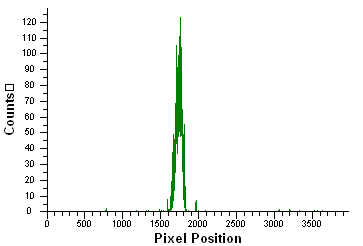


**Supplemental Fig. S5.** Radio-TLC analysis of precursors.

| Day | 10 | 14 | 21 | 24 | 28 | 31 | 38 | 45 | 52 |
| --- | --- | --- | --- | --- | --- | --- | --- | --- | --- |
| [^211^At]PSMA5 No. 1 | (±) | 30 (1+) | 30 (1+) | (±) | (±) | (-) | (-) | (±) | (±) |
| [^211^At]PSMA5 No. 2 | 30 (1+) | (±) | (±) | (-) | (-) | (-) | (-) | (-) | (-) |
| [^211^At]PSMA5 No. 3 |  | (±) | 30 (1+) | (-) | (-) | (-) | 30 (1+) | (-) | (-) |
| [^211^At]PSMA6 No. 1 | (±) | (-) | (-) | (±) | (±) | (-) | (-) | (-) | (-) |
| [^211^At]PSMA6 No. 2 | (±) | (±) | 30 (1+) | 100 (2+) | (-) | (±) | 30 (1+) | 30 (1+) | (±) |
| [^211^At]PSMA6 No. 3 | (±) | (±) | (±) | (±) | (-) | 30 (1+) | (-) | (±) | (±) |
| Control No. 1 | 30 (1+) | (±) | (-) | (-) | (-) | 30 (1+) | 30 (1+) | (-) | (-) |
| Control No. 2 | (±) | 30 (1+) | (±) | (±) | (-) | (-) | 30 (1+) | 30 (1+) | (-) |
| Control No. 3 |  | (±) | (±) | (±) | (-) | (-) | (-) | 30 (1+) | (-) |
| Control No. 4 |  | (±) | (±) | (±) | (±) | (±) | (±) | 30 (1+) | (±) |

**Supplementary Table S1.** Results of urine tests of protein (mg/dl) until 8 weeks after the administration of [^211^At]PSMA5 (0.4 MBq) and [^211^At]PSMA6 (0.4 MBq) in ICR mice.
